# Comparison of Volumetric Quantitative PET Parameters Before and After a CT-Based Elastic Deformation on Dual-Time 18FDG-PET/CT Images: A Feasibility Study in a Perspective of Radiotherapy Planning in Head and Neck Cancer

**DOI:** 10.3389/fmed.2022.831457

**Published:** 2022-02-11

**Authors:** Meriem Maajem, Jean-Christophe Leclère, David Bourhis, Valentin Tissot, Nicolas Icard, Laëtitia Arnaud, Romain Le Pennec, Gurvan Dissaux, Dorothy M. Gujral, Pierre-Yves Salaün, Ulrike Schick, Ronan Abgral

**Affiliations:** ^1^Department of Nuclear Medicine, Brest University Hospital, Brest, France; ^2^Department of Oto-Rhino-Laryngology, Brest University Hospital, Brest, France; ^3^European University of Brittany, UMR 1304 GETBO, IFR 148, Brest, France; ^4^Department of Radiology, Brest University Hospital, Brest, France; ^5^Department of Nuclear Medicine, Saint-Brieuc Regional Hospital, Saint-Brieuc, France; ^6^Department of Radiotherapy, Brest University Hospital, Brest, France; ^7^Clinical Oncology Department, Imperial College Healthcare National Health Service (NHS) Trust, Charing Cross Hospital, London, United Kingdom; ^8^Department of Cancer and Surgery, Imperial College London, London, United Kingdom

**Keywords:** elastic registration, dual time 18FDG-PET/CT images, radiotherapy planning, head neck cancer, gradient based method

## Abstract

**Background:**

The use of 18FDG-PET/CT for delineating a gross tumor volume (GTV, also called MTV metabolic tumor volume) in radiotherapy (RT) planning of head neck squamous cell carcinomas (HNSCC) is not included in current recommendations, although its interest for the radiotherapist is of evidence. Because pre-RT PET scans are rarely done simultaneously with dosimetry CT, the validation of a robust image registration tool and of a reproducible MTV delineation method is still required.

**Objective:**

Our objective was to study a CT-based elastic registration method on dual-time pre-RT 18FDG-PET/CT images to assess the feasibility of PET-based RT planning in patients with HNSCC.

**Methods:**

Dual-time 18FDG-PET/CT [whole-body examination (wbPET) + 1 dedicated step (headPET)] were selected to simulate a 2-times scenario of pre-RT PET images deformation on dosimetry CT. ER-headPET and RR-headPET images were, respectively, reconstructed after CT-to-CT rigid (RR) and elastic (ER) registrations of the headPET on the wbPET. The MTVs delineation was performed using two methods (40%SUVmax, PET-Edge). The percentage variations of several PET parameters (SUVmax, SUVmean, SUVpeak, MTV, TLG) were calculated between wbPET, ER-headPET, and RR-headPET. Correlation between MTV values was calculated (Deming linear regression). MTVs intersections were assessed by two indices (OF, DICE) and compared together (Wilcoxon test). Additional per-volume analysis was evaluated (Mann-Whitney test). Inter- and intra-observer reproducibilities were evaluated (ICC = intra-class coefficient).

**Results:**

36 patients (30M/6F; median age = 65 y) were retrospectively included. The changes in SUVmax, SUVmean and SUVpeak values between ER-headPET and RR-headPET images were <5%. The variations in MTV values between ER-headPET and wbPET images were −6 and −3% with 40%SUVmax and PET Edge, respectively. Their correlations were excellent whatever the delineation method (R^2^ > 0.99). The ER-headPET MTVs had significant higher mean OF and DICE with the wbPET MTVs, for both delineation methods (*p* ≤ 0.002); and also when lesions had a volume > 5cc (excellent OF = 0.80 with 40%SUVmax). The inter- and intra-observer reproducibilities for MTV delineation were excellent (ICC ≥ 0.8, close to 1 with PET-Edge).

**Conclusion:**

Our study demonstrated no significant changes in MTV after an elastic deformation of pre-RT 18FDG-PET/CT images acquired in dual-time mode. This opens possibilities for HNSCC radiotherapy planning improvement by transferring GTV-PET on dosimetry CT.

## Introduction

Head and neck squamous cell carcinoma (HNSCC) is the 6th most common cancer, with around 800,000 new cases worldwide in 2015 (1, 2). These tumors confer a poor prognosis, with a 5-year survival rate <50% and nearly 432,000 annual global deaths (3), probably because of diagnosis at an advanced stage in 2/3 of cases. Radiotherapy (RT) ± concomitant chemotherapy, as well as surgery, is the standard curative treatment of locally advanced tumors (4, 5). However, despite the improvement in treatment modalities and RT techniques, particularly the advent of intensity-modulated radiotherapy (IMRT), the locoregional failure rate remains high (4, 6).

18F-flurorodesoxyglucose (18FDG) positron emission tomography (PET/CT) is currently recommended for the pre-treatment staging of stage III-IV (T3-4, N1-3) HNSCC to look for distant metastases (7). Its indication remains optional for the early stages although its excellent performance for the diagnosis of lymph node involvement or of synchronous primary cancer have been demonstrated (8–10). In addition, in a recent study conducted on 477 patients, the management plan including RT was altered for 20% after implementation of 18FDG-PET/CT in the initial work up, whatever the stage (11).

However, the use of 18FDG-PET/CT for delineating the gross tumor volume (GTV) in radiotherapy (also called metabolic tumor volume MTV) is not included in current recommendations although its interest for the radiotherapist is of clinical relevance (12, 13). Some studies have shown that the GTV delineation in PET improved inter- and intra-observer reproducibility compared to CT (14), while having a better concordance with the macroscopic tumor volume. Furthermore, it has been proven that the GTV-PET was significantly lower than the GTV-CT (15–18), potentially leading to a reduction in organ at risk (OAR) toxicity (19).

For its use in clinical routine, two current issues remain to be resolved. Ideally, RT planning should be done on an 18FDG-PET combined with a dedicated contrast-enhanced computed tomography (ceCT) in the same treatment position with restraint method (thermoformed mask, personalized head support) and laser positioning (20) for achieving dosimetry using a consensual and reproducible delineation method. However, 18FDG-PET/CT for tumor staging is most often performed prior to the decision for RT curative treatment, with non-optimal conditions for treatment planning. In this situation, it is essential to integrate the PET data into the dosimetry ceCT after image registration, potentially modifying the GTV-PET. Indeed, the anatomical relationships between the different structures in the head and neck area strongly depend on the patient position. Moreover, there is currently no consensus on the PET delineation method as reported by the AAPG task group 211 in 2018, even if IA approaches based on convolutional neural networks are very promising (21, 22). Nevertheless, many approaches actually implemented in commercially softwares have been studied in head and neck cancers, such as those based on a relative threshold of SUVmax = 40% (23), on an adaptive threshold to local background noise (24) or on image gradients (25). This last-mentioned method, developed by Geets et al. has been reported to allow delineating a GTV-PET as close as possible to the macroscopic tumor volume.

Recent software currently proposes a CT-to-CT elastic image registration method and incorporates a gradient-based segmentation tool, called PET-Edge. To study its feasibility in RT planning and to simulate a 2-times scenario of an elastic registration of the pre-treatment PET/CT images on the dosimetry ceCT performed in a different position, we selected PET/CT performed in a “dual-time” mode, including a whole-body examination (wbPET) with arms raised followed by a dedicated head and neck acquisition (headPET) with arms down.

The objectives of this study were: (i) to evaluate the percentage change of usual (SUVmax, SUVmean, SUVpeak) and volumetric (MTV, TLG) quantitative parameters after rigid (RR) and elastic registration (ER) of headPET images on wbPET images; (ii) to compare the intersections between MTVs delineated on both headPET registered with RR or ER methods and MTVs delineated on wbPET; (iii) and to analyze inter- and intra-observer reproducibility for MTVs delineation with the respective 40%SUVmax and PET-Edge methods.

## Materials and Methods

### Population

Consecutive patients with HNSCC referred to a single institutional Nuclear Medicine department for a pre-treatment 18FDG-PET/CT from February 2017 to March 2019 were retrospectively analyzed.

Patients were included if their PET examination was performed in a dual-time acquisition mode.

The exclusion criteria were as follows: poor positioning of the arms, lesion of interest out of the wbPET field of view (FOV), absence of hypermetabolism over target tumor, or multimetastatic disease.

Usual clinical and histopathological data [age, sex, tumor location, World Health Organization (WHO) classification and American Joint Cancer Classification (AJCC) stage] were collected (26, 27).

This study was approved by an Ethics Committee (CEMEN 2021-03).

### 18FDG-PET/CT Imaging

All exams were performed on the same Biograph TruePoint™ 64 PET/CT system (Siemens healthineers^®^, Erlangen, Germany) after injection of 3 MBq/Kg of 18FDG (Curium^®^, Saclay, France).

Dual-time acquisition consisted of an initial whole-body acquisition (2 min 30 per step) in dorsal decubitus with arms raised (wbPET) ~1 h after tracer injection, followed by a 1-step acquisition (5 min) centered on the head and neck area with arms down (headPET).

PET images were reconstructed with an iterative OSEM 3D method and corrected for random coincidences, scatter and attenuation using the CT scan. The reconstructed images parameters were as follows: wbPET (cutting thickness = 5 mm; matrix = 168^2^; voxel size = 4.1 × 4.1 × 5 mm) and headPET (cutting thickness = 3 mm; matrix = 256^2^; voxel size = 2.7 × 2.7 × 3 mm).

All PET data were anonymized and then analyzed blindly by 2 nuclear medicine physicians to assess inter-observer reproducibility (MM, RA).

To assess intra-observer reproducibility, a second analysis by the most experienced operator was performed 3 months later on a third of the randomly selected cohort.

### Image Processing

Preliminary image processing was performed on Mim software (v7, Cleveland, USA) in two steps.

The first step involved the creation of the fused series with the VoxAlign Engine^®^ tool. After a rapid rigid registration (RR) between the wbCT and the headCT resulting from the two respective PET/CT acquisitions, a precise RR spatially limited to the primary location area to be analyzed was performed. An elastic registration (ER) was then performed, allowing a superimposition of different anatomical structures. The registration matrix resulting from these two deformations were applied to the headPET images, allowing acquisition of two new series of PET images with rigid (RR-headPET) and elastic deformation (ER-headPET) for each patient.

The second step consisted of delineating the metabolic tumor volume (MTV, in cc) on the wbPET, the ER-headPET and RR-headPET using 2 different methods: a relative thresholding method (40%SUVmax) and a gradient-based method (PET-Edge™).

### Data Analysis

The extraction of the usual quantitative PET parameters was carried out on the 3 series of wbPET, ER-headPET and RR-headPET images, delineated with the 2 methods (40%SUVmax and PET-Edge). The values of SUVmax, SUVmean, and SUVpeak were measured within the MTV and then the Total Lesion Glycolysis was calculated with the formula TLG = MTV × SUVmean.

The one-to-one intersection of A and B volumes from the different series of images wbPET, ER-headPET and RR-headPET was evaluated by two methods: the overlap fraction (OF) index given by the formula OF (A, B) = (AnB)/B and the DICE coefficient given by the formula DICE (A, B) = (2 × AnB)/(A + B). The values of each parameter vary between 0 if the volumes A and B are completely disjointed and 1 if they match perfectly in size, shape and location.

### Statistical Analysis

The one-to-one mean ± SD relative changes (%) of the usual (SUVmax, SUVmean, SUVpeak) and volumetric (MTV, TLG) PET parameters according to the different reconstructed wbPET, ER-headPET, and RR-headPET images were calculated.

The correlation between MTV values was calculated using a Deming linear regression test with a R^2^ measurement.

The OF and DICE mean ± SD values (wbPET vs. ER-headPET or RR-headPET) were compared using the Wilcoxon test. A difference between the OF and DICE coefficient values according to the lesion volume, arbitrarily defined with a threshold of MTV = 5cc measured on the wbPET, was evaluated using the Mann-Whitney test.

The inter- and intra-observer reproducibility were evaluated by calculating the intra-class coefficient (ICC).

The Landis and Koch scale was used to assess the quality of the intersection and the reproducibility: 0–0.2 (very low); 0.2–0.4 (weak); 0.4–0.6 (moderate); 0.6–0.8 (strong); 0.8–1 (excellent) (28).

The significance threshold was given by a *p*-value of < 0.05.

All statistical analyzes were performed with XLStat 2019 software (Addinsoft^®^, Paris, France).

## Results

Between February 2017 to March 2019, 55 patients with HNSCC underwent a 18FDG-PET/CT scan in a dual-time acquisition mode for the initial assessment of their disease.

Among them, 19 were excluded from the analysis for the following reasons: poor positioning of the arms in 2 cases (both acquisitions with arms down), primary location out of the wbPET FOV in 13 cases, no hypermetabolism over target lesion in 3 cases, multi-metastatic disease in 1 case.

**Figure 1:**
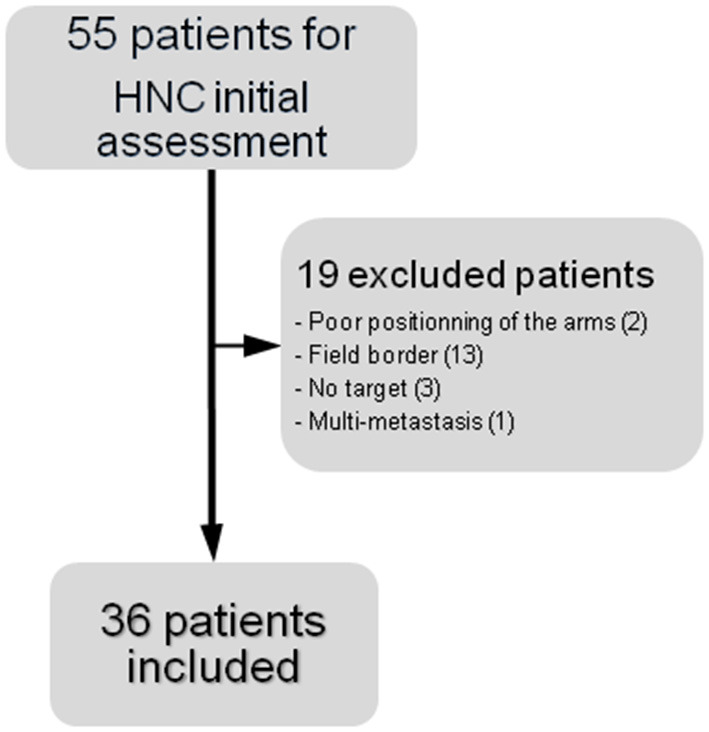
Flow chart of the study.

Therefore, 36 patients were retrospectively included in this study (Figure 1).

### Patient Characteristics

The median age at diagnosis was 65 years and the sex ratio M/F was 3.3. Primary lesions were mainly located in the oropharynx (*n* = 15) and concerned cervical lymph node carcinoma from unknown primary (CUP) in 4 cases (11%). The majority were locally advanced cancers (39% of T4 lesions and 72% of AJCC stage IV).

Table 1 summarizes the characteristics of the population.

**Table 1:** Patients characteristics.

**Characteristics**	**No of patients** **(***n*** = 36)**
**Age (mean ±Sd)**	61 ± 9
**Sex (male/female)**	30/6
**Tumor location** ***n*** **(%)**
Oral cavity	5 (14)
Oropharynx	15 (42)
Hypopharynx	7 (20)
Larynx	4 (11)
Nasopharynx	1 (3)
CUP	4 (11)
**AJCC** ***n*** **(%)**
I	2 (6)
II	1 (6)
III	7 (20)
IV	26 (72)
**WHO** ***n*** **(%)**
Tx	4 (11)
T1	6 (17)
T2	8 (22)
T3	4 (11)
T4	14 (39)

### 18FDG-PET/CT Analysis

The wbPET acquisition was performed on average of 63 ± 7 min after injection of 3.0 ± 0.1 MBq of 18FDG.

The headPET acquisition started on average of 18 ± 2 min after the wbPET.

### Variation of Quantitative Parameters

Table 2 shows the relative changes of quantitative parameters between ER-headPET and RR-headPET and wbPET reconstructed images.

**Table 2:** Mean change (in %) ± SD of quantitative parameters.

	**ER-headPET**		**ER-headPET**	
	**vs. wbPET**		**vs. RR-headPET**	
**Parameters**	**40%SUVmax**	**PET-Edge**	**40%SUVmax**	**PET-Edge**
SUVmax	21 ± 17	21 ± 18	4 ± 4	3 ± 3
SUVmean	11 ± 7	12 ± 18	−1 ± 4	0 ± 1
SUVpeak	14 ± 10	14 ± 10	0 ± 0	0 ± 0
TLG	5 ± 24	5 ± 38	−6 ± 11	−8 ± 13
MTV	−6 ± 18	−3 ± 37	−6 ± 12	−8 ± 13

#### ER-HeadPET vs. WbPET

The mean changes of SUVmax, SUVmean, and SUVpeak were +21%, +11% and +14%, respectively, with 40%SUVmax method; and +21%, +12%, and +14% with PET-Edge method.

The mean changes of TLG were +5% regardless of the delineation method.

The mean changes of MTV were −6% and −3% with 40%SUVmax and PET-Edge methods, respectively.

#### ER-HeadPET vs. RR-HeadPET

The mean changes of SUVmax, SUVmean, and SUVpeak were +4%, +1%, and <1%, respectively, with 40%SUVmax method; and +3%, <1%, and +1% with PET-Edge method.

The mean changes of both MTV and TLG were −6% and −8%, respectively, with 40%SUVmax and PET-Edge methods.

### Comparison of Volumes

**Figure 2:**
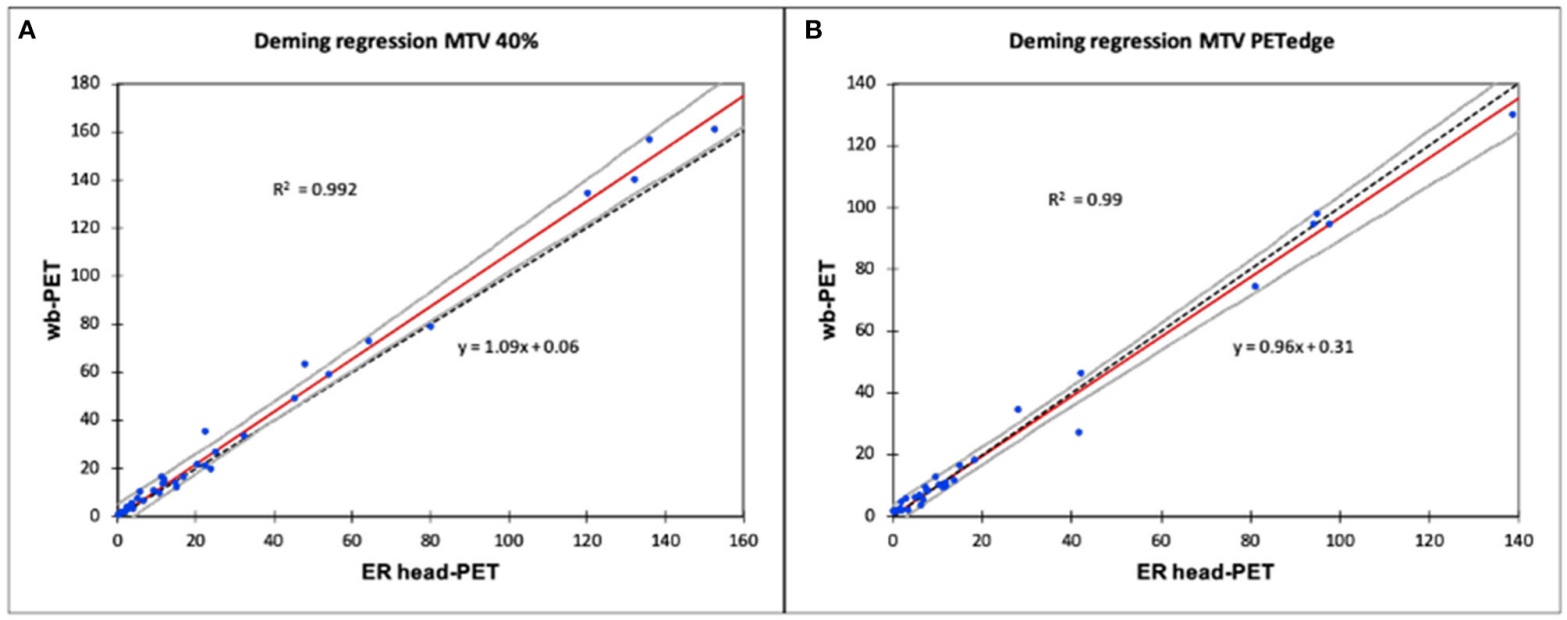
Relationship between MTV delineated on ER-headPET and wbPET using a Deming linear regression. **(A)** Deming regression MTV 40%. **(B)** Deming regression MTV PETedge.

Deming regressions showed an excellent linear relationship between the MTV volumes delineated on the ER-headPET and wbPET images with both 40%SUVmax (R^2^ = 0.992) and PET-Edge (R^2^ = 0.99) methods (Figures 2A,B).

The confidence interval around the estimated line included y = x, except from the 4 largest lesions (>100cc) delineated with the 40%SUVmax method.

### MTV Intersections

#### According to Registration Method

The mean OF and DICE indices of intersection between the MTV delineated on wbPET with, respectively, those on the ER-headPET and the RR-headPET are shown in Table 3.

**Table 3:** Mean OF and DICE indices between wbPET and headPET volume delineations according to RR and ER methods.

	**Overlap fraction**			**DICE coefficient**		
**Method**	**RR-headPET**	**ER-headPET**	***p*** **-value**	**RR-headPET**	**ER-headPET**	***p*** **-value**
	**(***n*** = 36)**	**(***n*** = 36)**		**(***n*** = 36)**	**(***n*** = 36)**	***p*** **-value**
40%SUVmax	0.58 ± 0.26	0.76 ± 0.13	<0.0001^*^	0.57 ± 0.25	0.70 ± 0.15	<0.0001^*^
PET-Edge	0.53 ± 0.26	0.69 ± 0.19	<0.0001^*^	0.53 ± 0.26	0.62 ± 0.18	0.002^*^

* *Significancy of result*.

**Figure 3:**
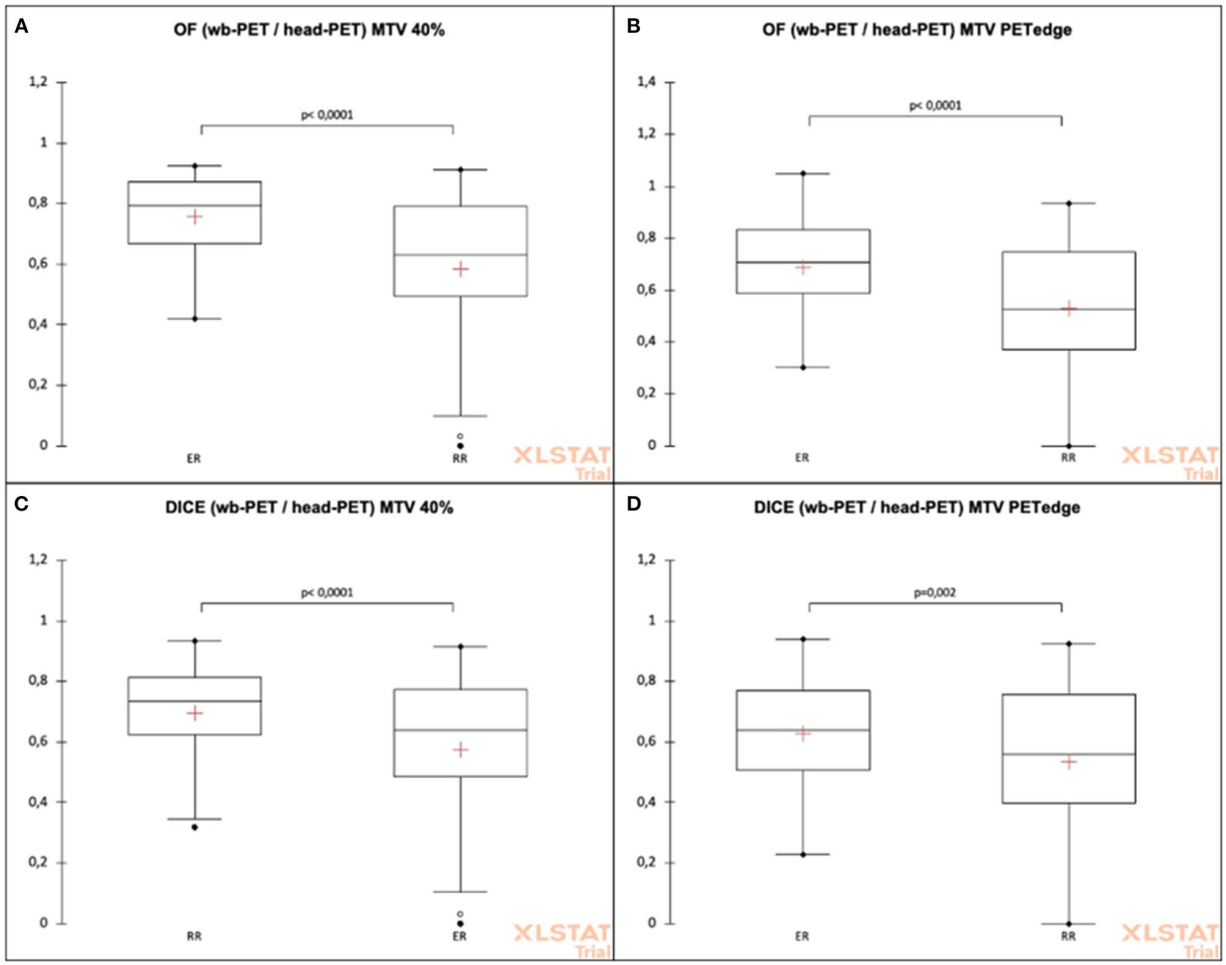
OF and DICE coefficients comparison between headPET and wbPET MTVs according to the registration (elastic or rigid) and the delineation methods (40%SUV max or PET-Edge). **(A)** OF (wb-PET/head-PET) MTV 40%. **(B)** OF (wb-PET/head-PET) MTV PETedge. **(C)** DICE (wb-PET/head-PET) MTV 40%. **(D)** DICE (wb-PET/head-PET) MTV PETedge.

The ER-headPET MTV had significantly higher mean OF indices than the RR-headPET MTV with the wbPET MTV for both 40%SUVmax (OF = 0.76 ± 0.13 vs. 0.58 ± 0. 26, *p* < 0.0001) and PET-Edge (OF = 0.70 ± 0.15 vs. 0.57 ± 0.25, *p* < 0.0001) methods (Figures 3A,B).

The ER-headPET MTV had significantly higher mean DICE indices than the RR-headPET MTV with the wbPET MTV for both 40%SUVmax (DICE = 0.69 ± 0.19 vs. 0.53 ± 0.26, *p* < 0.0001) and PET-Edge (DICE = 0.62 ± 0.18 vs. 0.53 ± 0.26, *p* = 0.002) methods (Figures 3C,D).

#### According to Tumor Volume

The mean OF and DICE indices of intersection between the MTV according to tumor volume are shown in Table 4.

**Table 4:** Mean OF and DICE indices between wbPET and ER-headPET volume according to delineation method and to tumor volume (cut-off 5cc).

	**Overlap fraction**			**DICE coefficient**		
**Cut off**	**≤5cc**	**>5cc**	***p*** **-value**	**≤5cc**	**>5cc**	***p*** **-value**
40%max (*n* = 8 vs. 28)	0.61 ± 0.14	0.80 ± 0.10	0.001^*^	0.52 ± 0.14	0.75 ± 0.11	0.0002^*^
PET-Edge (*n* = 11 vs. 25)	0.63 ± 0.24	0.71 ± 0.15	0.183	0.44 ± 0.13	0.71 ± 0.14	<0.0001^*^

* *Significancy of result*.

**Figure 4:**
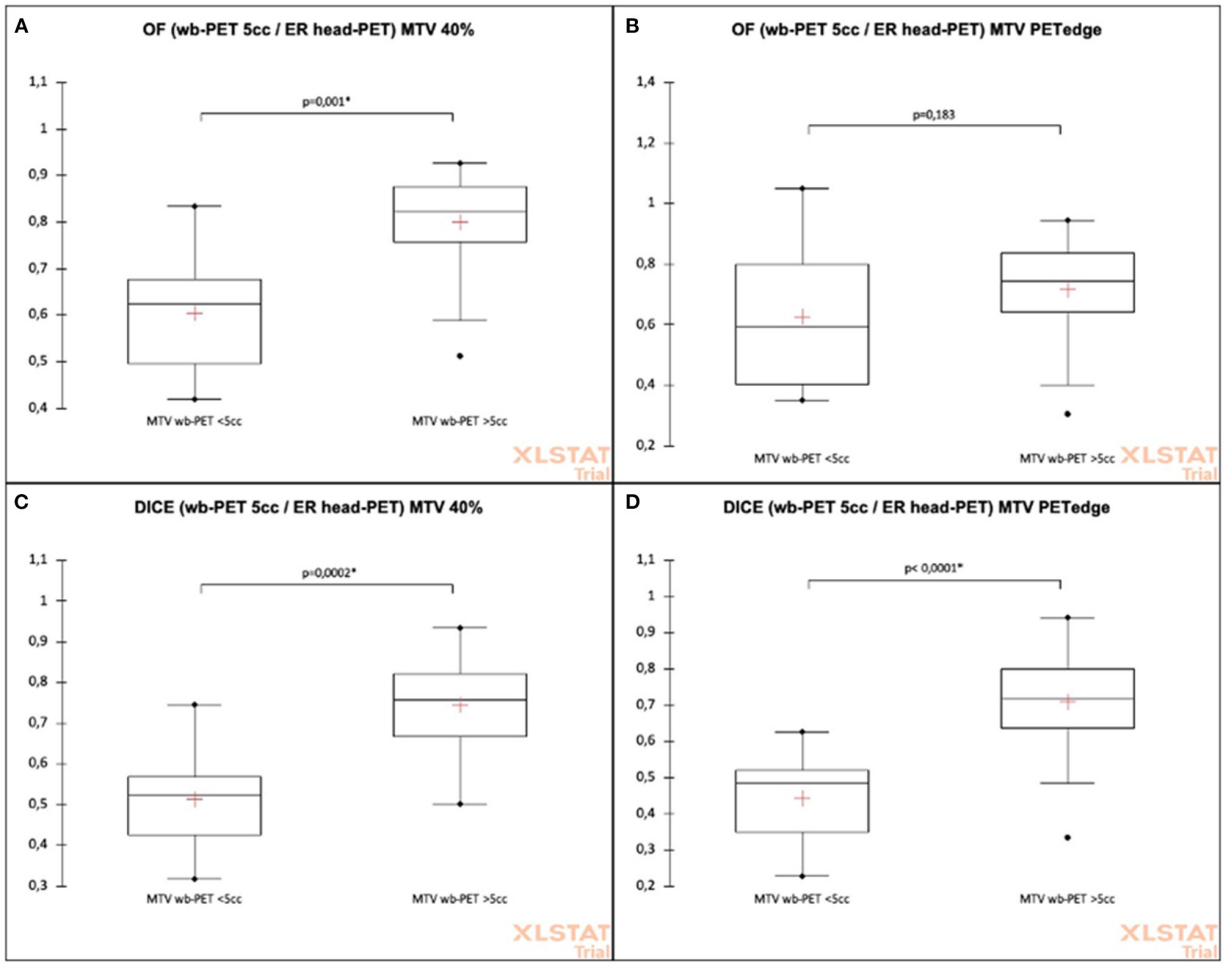
Comparison of OF and DICE intersection indices between wbPET and ER-headPET volumes according to delineation method and to tumor volume (cut-off 5cc). **(A)** OF (wb-PET 5cc/ER head-PET) MTV 40%. **(B)** OF (wb-PET 5cc/ER head-PET) MTV PETedge. **(C)** DICE (wb-PET 5cc/ER head-PET) MTV 40%. **(D)** DICE (wb-PET 5cc/ER head-PET) MTV PETedge.

The ER-headPET MTV had significantly higher mean OF indices with the wbPET MTV when lesions had a volume > 5cc for 40%SUVmax (mean OF = 0.80 ± 0.10 vs. 0.61 ± 0.14, *p* = 0.001) method. This difference was not significant for PET-Edge (OF = 0.71 ± 0.15 vs. 0.63 ± 0.24, *p* = 0.183) method (Figures 4A,B).

The ER-headPET MTV had significantly higher mean DICE indices with the wbPET MTV when lesions had a volume > 5cc for both 40%SUVmax (DICE = 0.75 ± 0.11 vs. 0.52 ± 0.14, *p* = 0.0002) and PET-Edge (DICE = 0.71 ± 0.14 vs. 0.44 ± 0.13, *p* = 0.183) methods (Figures 4C,D).

**Figure 5:**
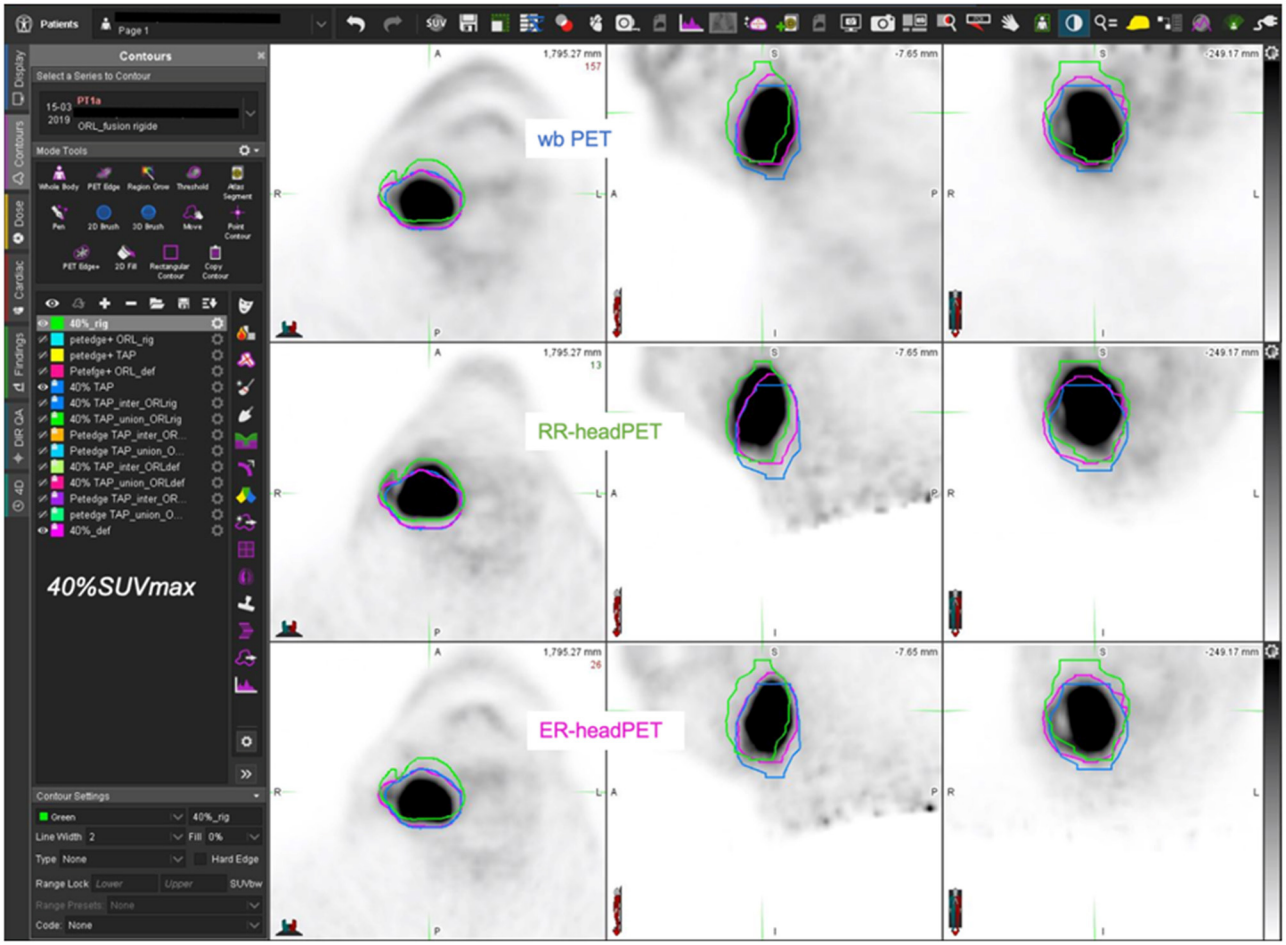
59-year-old man with T4 N1 M0 oropharyngeal SCC (AJCC stage IV) – estimated MTV = 49.0cc. wbPET MTV (=V1 in blue), RR-headPET MTV (=V2 in green) and ER-headPET MTV (=V3 in pink) delineated with 40%SUVmax segmentation method reported on the wbPET (top row), RR-headPET (middle row) and ER-headPET (bottom row) images in axial (left column), sagittal (center column) and coronal (right column) views. OF (V3, V1) = 86.6% vs. OF (V2, V1) = 59.4%; DICE (V3, V1) = 83.2% vs. DICE (V2, V1) = 60.5%.

**Figure 6:**
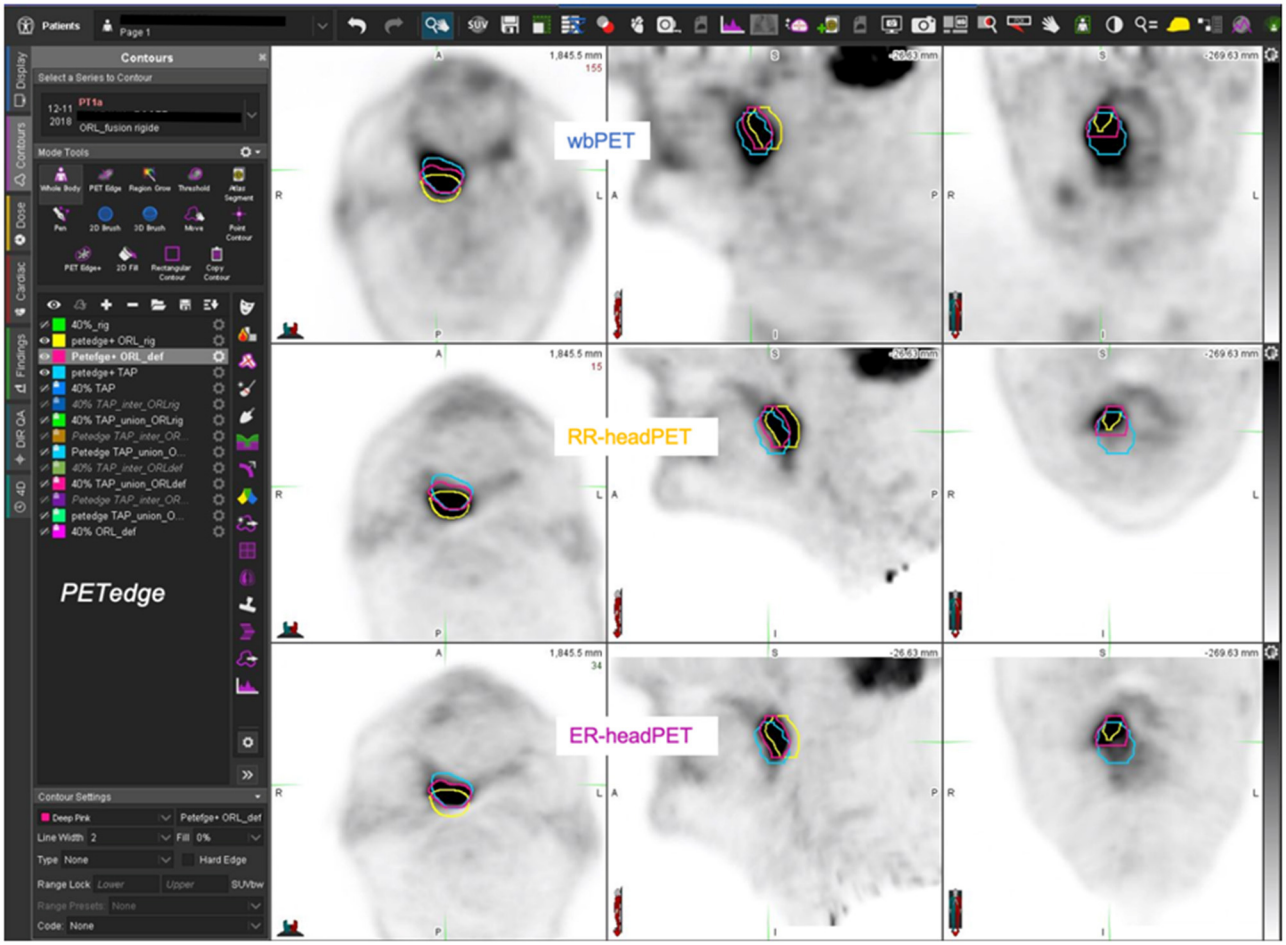
77-year-old man with T3 N1 M0 oropharyngeal SCC (AJCC III stage) – estimated MTV = 16.3cc. wbPET MTV (=V1 in blue), RR-headPET MTV (=V2 in yellow) and ER-headPET MTV (=V3 in pink) delineated with PET-Edge segmentation method reported on the wbPET (top row), RR-headPET (middle row) and ER-headPET (bottom row) images in axial (left column), sagittal (center column) and coronal (right column) views. OF (V3, V1) = 60.1% vs. OF (V2, V1) = 37.9%; DICE (V3, V1) = 55.3% vs. DICE (V2, V1) = 37.6%.

**Figure 7:**
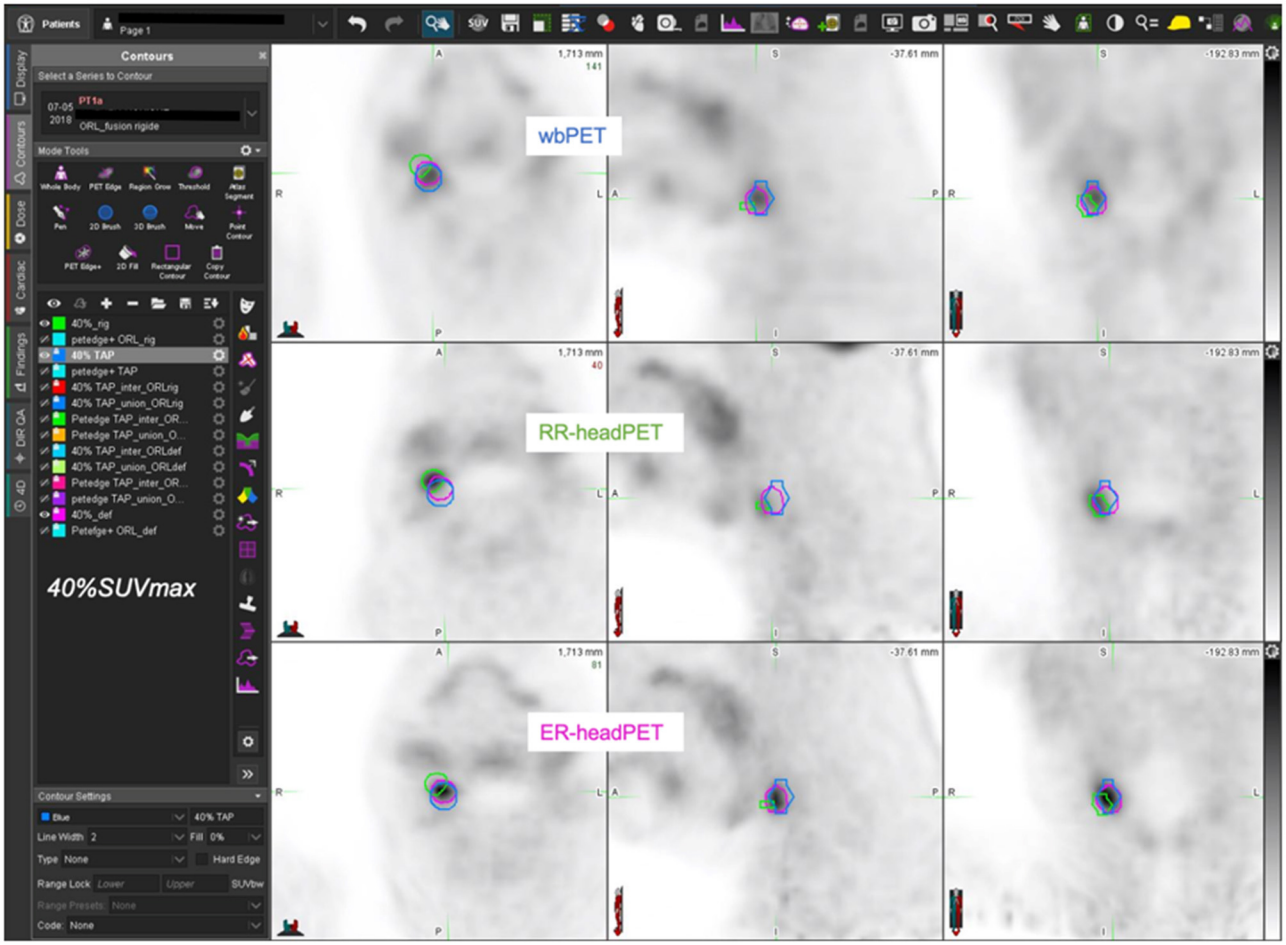
63-year-old man with T1 N1 M0 oropharyngeal SCC (AJCC stage III) – estimated MTV = 0.9cc. wbPET MTV (=V1 in blue), RR-headPET MTV (=V2 in green) and ER-headPET MTV (=V3 in pink) delineated with 40%SUVmax segmentation method reported on the wbPET (top row), RR-headPET (middle row) and ER-headPET (bottom row) images in axial (left column), sagittal (center column) and coronal (right column) views. OF (V3, V1) = 71.2% vs. OF (V2, V1) = 2.9%; DICE (V3, V1) = 74.5% vs. DICE (V2, V1) = 3.1%.

Figures 5–7 show three different examples of OF and DICE results according to the images registration and the MTV delination methods used.

### Reproducibility of MTV Delineation

The inter- and intra-observer reproducibility analysis for the MTV delineation according to the 40%SUVmax and PET-Edge methods are shown in Table 5.

**Table 5:** Inter- and intra-observer reproducibility (ICC) on MTV delineation.

	**wbPET**		**RR-headPET**		**ER-headPET**	
**Reproducibility**	**40%SUVmax**	**PET-Edge**	**40%SUVmax**	**PET-Edge**	**40%SUVmax**	**PET-Edge**
Inter-observer	0.80	0.99	0.81	0.99	0.81	0.98
Intra-observer	0.90	0.99	0.89	0.99	0.90	0.99

The inter-observer reproducibility for the MTV delineation on each series of images was excellent for both 40%SUVmax (ICC from 0.80 to 0.81) and PET-Edge (ICC 0.98–0.99) methods.

The intra-observer reproducibility for the MTV delineation on each series of images was excellent for both 40%SUVmax (ICC 0.89–0.90) and PET-Edge (ICC 0.99) methods.

## Discussion

To the best of our knowledge, this study is the first to assess the feasibility of applying an elastic registration on a dual-time PET-CT examination to simulate radiotherapy planning for head and neck (HN) cancers using an exported GTV-PET to the dosimetry CT.

First, we found a significant increase in the values of SUVmax, SUVmean, and SUVpeak between the ER-headPET and wbPET image series, logically explained by the mean delay of 18 ± 2 min between these two-times acquisitions. Current guidelines recommend acquiring PET images 60 ± 5 min after injection of 18FDG, in particular for reasons of reproducibility and comparability in the event of repeated examinations in the same patient (7, 29). However, the 18FDG uptake in tumors is a dynamic process, reaching a plateau phase at varying times (from 30 min to several hours), depending on their histological type (30, 31). This 20% increase in SUVmax reported in our results is related to the fact that the maximum absorption of 18FDG in squamous carcinoma cell (SCC) occurs more than 1 h after tracer administration. Indeed, in a dual-time PET study including 66 HNSCC, Abgral et al. found an increase in SUVmax on average from 7.2 to 9.2 between 1 and 2 h post-injection of 18FDG (32). Consequently, recent literature suggested applying this delayed protocol to improve diagnostic performance of 18FDG-PET/CT in head and neck cancers (33–36).

Conversely, metabolic tumor volumes (MTV) delineated using a relative threshold of SUVmax were only slightly affected in our results (around 5% maximum). This suggests that dynamic uptake profile of each voxel in the tumor remains identical over time. In another series of 80 PET/CT scanned in a 1–2 h dual-time mode, Abgral et al. also showed a deltaMTV <5% of primary head and neck lesions. Finally, the TLG value reflecting the metabolic tumor burden was logically affected by this dual-time acquisition in our series, since it is by definition dependent on the SUVmean. However, TLG is more a prognostic indicator than a radiotherapy planning tool. However, an RT dose escalation decision may be considered in cases of high TLG value (37).

Our comparison of the RR-headPET and ER-headPET images showed a slight change in the values of static (<5%) and volumetric (6–8%) quantitative parameters, thus demonstrating the feasibility of transferring PET data by elastic registration onto a dosimetry CT, without significant loss of information. Our Deming regression analysis confirmed this comparability of volumes with excellent linearity coefficients (R^2^ > 0.99) whatever the segmentation method used. Only a relative MTV underestimation after ER was observed using the 40%SUVmax delineation method (but not PET-Edge) from the 4 lesions with a volume > 100cc. This could be related to the difference in the reconstruction matrix parameters of the wbPET and headPET image series in our study.

In this cohort, we chose to compare two segmentation methods widely used to assess the metabolic tumor volume of HNSCC. The 40%SUVmax relative thresholding method, having the advantage of being freely available on the different image post-processing software from PET manufacturers, has largely demonstrated prognostic evaluation of head and neck cancers (38). Thus, by comparing different relative thresholds of SUVmax (30, 40, 50%), Abgral et al. showed that 40%SUVmax was the most relevant to predict overall survival (OS) and progression-free survival (PFS) in 80 patients treated for head and neck cancer (39). The same group confirmed that an MTV > 6.7cc delineated with the 40%SUVmax method was an independent predictive factor of poor PFS in a cohort of 70 HNSCC (32). The PET-Edge method, initially developed by Geets et al. (25), is based on PET image gradients and has also often been used in series involving HNSCC. In a cohort of 45 tumors of the oral cavity and oropharynx, Dibble et al. showed that MTVs delineated with PET-Edge had a greater prognostic impact than MTVs delineated with the 38%SUVmax, 50%SUVmax, and 60%SUVmax methods (40). Geets et al. suggested in 2 studies conducted on pharyngolaryngeal tumors that pre- and per-treatment planning in IMRT by GTV-PET using the PET-Edge method allowed a significant reduction in the dose to organs-at-risk (parotid glands, bone marrow spinal cord) compared to GTV-CT, while having an identical tumor control rate. They also highlighted the possibility of a PET-based dose escalation in RT (19, 41). Finally, apart from the head and neck region, Wanet et al. showed in a comparative series that this gradient-based method was the most efficient and robust (vs. GTV-PET 40%SUVmax, 50% SUVmax, Daisne and GTV-CT) to assess the real histopathological tumor volume of 10 resected T1-T2 bronchopulmonary cancers (42). The Daisne method, using an adaptive SUVmax threshold dependent on the background noise surrounding the tumor, also showed a good correlation between the GTV-PET and the macroscopic MTV, close to that found with PET-Edge (42). However, this method remains more tedious and requires a phase of calibration of the machine, not possible in our retrospective study (24).

Furthermore, our analysis of inter- and intra-observer variability showed an excellent reproducibility for the MTV delineation on each series of PET images according to our two studied methods. In addition, values of concordance were close to perfect agreement (ICC = 1) with PET-Edge, presumably because it is an automatic method requiring minimal user-input. These results are in agreement with the data in the literature. In a study of 10 head and neck cancers delineated by 8 different operators, Breen et al. showed excellent inter- and intra-observer reproducibility, even with a normally more subjective visual analysis method (ICC = 0.85 and 0.91, respectively) (14). In a more recent series, assessing the prognostic value of pre-therapeutic radiomic analysis in PET for 43 HNSCC, Guezennec et al. also found excellent interobserver reproducibility in the preliminary MTV delineation step with the 40%SUVmax (ICC = 0.99), Daisne (ICC = 0.99) and PET-Edge (ICC = 0.88) methods (43). These results support the idea of using 18FDG-PET/CT for radiotherapy planning, particularly as it has clearly demonstrated a decrease in inter- and intra-observer variability for the delineation of GTV-PET compared to GTV-CT. However, this delineation should be done by an experienced operator (14). Indeed, in an inter-observer reproducibility study, Bontemps et al. found conformity indices (intersection/union of volumes) significantly higher for a senior/senior pair of operators with more than 5 years of practice (vs. senior/resident) for the MTV delineation of 30 HNSCC, regardless the 4 segmentation methods used (SUV 2.5, 40%SUVmax, 50%SUVmax, Daisne) (44).

In addition, our results showed a significant improvement in the intersection indices between the MTV headPET and wbPET after elastic (ER) vs. rigid (RR)registration, whatever the segmentation method used. At best, OF indices presented an increase of around 18% with the 40%SUVmax segmentation method (OF = 0.76 ± 0.13 vs. 0.58 ± 0.26, *p* < 0.0001). The recent “hotspots” concept developed in PET, assessing the correlation between the area of high tumor uptake and the preferential site of recurrence before considering a RT dose escalation, necessitates registration of pre-therapy (A) and post-therapy (B) images (45). In a recent study including 71 HNSCC recurrences, Truffault et al. showed a real benefit in the elastic registration of images especially when patients were not scanned in treatment position (NTP). Thus, using the 40%SUVmax method, the OF (A40nR40) of MTVs was significantly higher after ER than RR (*p* < 0.03) (46, 47).

Finally, we carried out a complementary analysis by lesions in two dichotomized subgroups with a threshold of MTV = 5cc delineated on the reference wbPET. The aim of this arbitrary choice was to dissociate T1 tumors with a diameter of <2 cm that are generally less dependent on precise RT delineation than more advanced lesions. Therefore, we demonstrated a significant difference after elastic registration in the intersections between the ER-headPET and wbPET MTVs, whatever the delineation method.

For example, the average OF index became excellent (OF = 0.80 ± 0.10) with 40%SUVmax delineation method in the MTV > 5cc subgroup. Nevertheless, these results remain imperfect and support the idea that PET exams before radiotherapy planning should be performed in the ideal RT treatment position with a personalized head support and a thermoformed mask. In the previous mentioned “hotspot” study, Truffault et al. also showed an impact in performing pre- and post-therapeutic PET scans in the treatment position (TP) to improve the intersection between MTVs. Thus, OF (A40nR40) changed from low to moderate agreement, increasing from 0.31 ± 0.13 (NTP) to 0.50 ± 0.22 (TP) with a trend toward significance (*p* = 0.094) (46). On the contrary, the average DICE index evolved from strong to moderate agreement in our subgroup of MTV <5cc, in particular with PET-Edge method (DICE = 0.44 ± 0.13). This result suggests the need for particular vigilance when delineating the smallest lesions on PET in the event of an RT decision. Nevertheless, these relative differences of intersection would probably be integrated into the usual margins imposed on the GTV of a small lesion, also often poorly identifiable on morphological imaging (48).

Our study has several limitations. First, this is a retrospective and single-center study. Second, our population is not homogeneous. Indeed, our cohort includes a majority of advanced stage IV patients at diagnosis, which nevertheless reflects real-life practice and constitutes a large proportion of the population for curative radiotherapy (3). Moreover, the frequency of primary oropharyngeal/oral cavity locations in our series shows an inversion of usual prevalence. However, it still remains difficult to precisely determine the primary site of a T4 tumor in such areas (1). In addition, we included 4 cervical carcinomas of unknown primary (CUP) and therefore performed MTV delineation on the lymph node. However, the presentation of HNSCC is often with nodes and tumor continuity whose limits are difficult to be assessed in imaging. In any case, cervical lymph nodes would have fully integrated into the GTV-PET for RT. Third, our intra-observer reproducibility analysis was performed by a single operator (the more experienced) but with a robust methodology, comprising the re-anonymization of a third of randomly selected exams for re-analysis 3 months later to avoid selection and memory bias (49). Finally, the reconstruction parameters of image series were not strictly comparable since the headPET is the result of a dedicated complementary acquisition step with a higher matrix definition than the wbPET series to improve diagnostic performance (50). This may partly explain the low variability on quantitative measurements.

## Conclusion

Our study opens possibilities for the use of PET in radiotherapy planning of head and neck cancers by transferring the GTV-PET onto the dosimetry CT after a CT-to-CT-based elastic image registration, without significant changes in static and volumetric PET parameters. We found good MTV intersection indices (more limited for T1 tumors) and excellent inter and intra-observer reproducibility, in particular with a gradient-based delineation method, and with 40%SUVmax by an experienced user.

Future prospective studies are warranted to investigate performing PET examination under optimal RT treatment conditions to further improve MTVs intersections.

## Data Availability Statement

The original contributions presented in the study are included in the article/supplementary material, further inquiries can be directed to the corresponding author/s.

## Ethics Statement

The studies involving human participants were reviewed and approved by Nuclear Medicine Research Ethics Committee (CEMEN). The patients/participants provided their written informed consent to participate in this study. Written informed consent was obtained from the individual(s) for the publication of any potentially identifiable images or data included in this article.

## Author Contributions

RA, MM, J-CL, and US are the guarantors of the paper. RA, DB, MM, and P-YS designed the study. J-CL, US, GD, and VT ensured inclusion of patients. NI and LA managed imaging procedures. RA, DB, and MM analyzed patient records. RA and MM analyzed the data. RA and RL realized statistics. DG revised English language. All authors contributed in drawing up the manuscript. All authors contributed to the article and approved the submitted version.

## Conflict of Interest

The authors declare that the research was conducted in the absence of any commercial or financial relationships that could be construed as a potential conflict of interest.

## Publisher's Note

All claims expressed in this article are solely those of the authors and do not necessarily represent those of their affiliated organizations, or those of the publisher, the editors and the reviewers. Any product that may be evaluated in this article, or claim that may be made by its manufacturer, is not guaranteed or endorsed by the publisher.
